# 826. HIV Infection and HPV Genotype Patterns among Young Women with Advanced Cervical Neoplasia in Davidson County, Tennessee

**DOI:** 10.1093/ofid/ofab466.1022

**Published:** 2021-12-04

**Authors:** Leahanne Giffin, Yuwei Zhu, Manideepthi Pemmaraju, Sheelah Blankenship, Emmanuel Sackey, Troy Querec, Elizabeth R Unger, Angela Cleveland, Julia Gargano, Jessica L Castilho

**Affiliations:** 1 Vanderbilt University Medical Center, Nashville, Tennessee; 2 Vanderbilt University, Nashville, Tennessee; 3 Centers for Disease Control and Prevention (CDC), Atlanta, Georgia; 4 Centers for Disease Control and Prevention, Atlanta, GA

## Abstract

**Background:**

Women living with HIV (WLWH) experience high rates of human papillomavirus (HPV) infection and increased risk of cervical cancer. High-risk HPV (HR-HPV) types 16/18 cause most cervical precancers and cancers in women with and without HIV. However, contributions of other HR-HPV types to cervical disease among WLWH are not fully understood. We compared CIN2+ cases (cervical intraepithelial neoplasia grade 2 or higher or adenocarcinoma in situ) and the association between non-16/18 HPV types among women with and without HIV.

**Methods:**

Davidson County, Tennessee, women aged 18-39 years with CIN2+ diagnosed between 2008-2016 with HPV genotyping were included. HIV status, demographics, and histology were abstracted from medical records. Neighborhood-level socioeconomic factors were derived from *Integrated Public Use Microdata Series.* Archived cervical tissue was tested for 37 HPV types to define CIN2+ cases negative for HPV 16/18, regardless of presence of other HR-HPV strains. Characteristics of women with CIN2+ and HPV typing patterns were compared between women with and without HIV using Wilcoxon and Chi-square tests. Logistic regression assessed the association of non-16/18 HPV types and HIV infection, adjusting for age, race, calendar year, insurance, HPV vaccination, and neighborhood socioeconomic factors (selected a priori).

**Results:**

Among 2,116 women included, 1,093 (52%) had neither HPV16 nor HPV18. Compared to women without HIV, the 27 WLWH included were more likely to be >30 years of age, Black race, and live in neighborhoods with higher measures of poverty (Table 1). HPV types did not statistically differ by HIV status, though WLWH had a higher number of HR-HPV types present (Table 2). HIV infection was not significantly associated with non-16/18 HPV type after adjusting for confounders (adjusted OR 0.86 [95%CI: 0.4-1.88]).

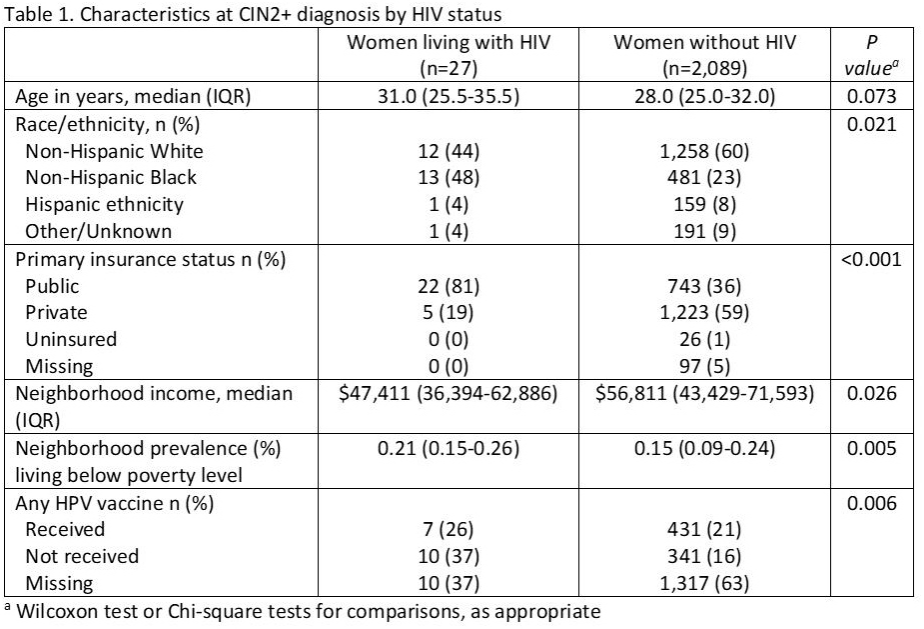

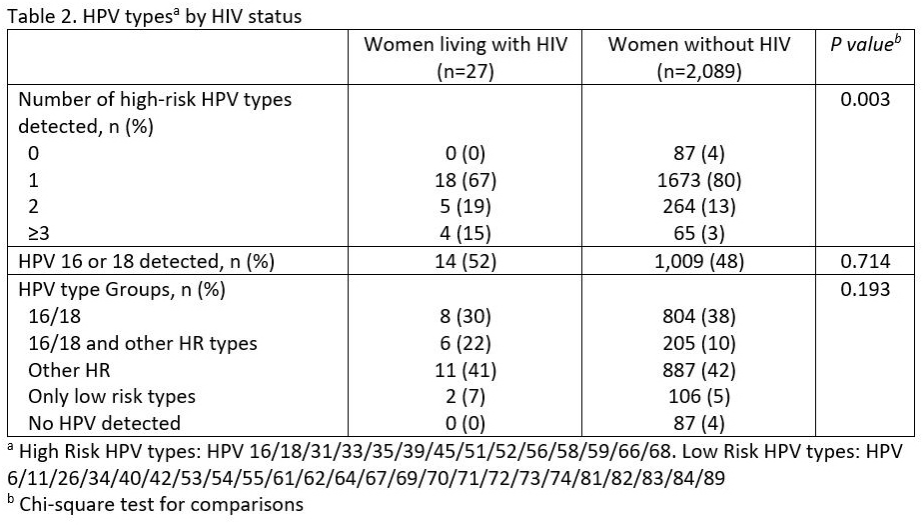

**Conclusion:**

Among women with CIN2+, HIV infection was not significantly associated with non-16/18 HPV types. However, WLWH had a higher number of high-risk HPV types detected. Our study was limited by the small number of WLWH included.

**Disclosures:**

**All Authors**: No reported disclosures

